# USP5‐C‐MAF Axis Regulates Autophagy‐Dependent Neuronal Ferroptosis in Spinal Cord Injury Therapeutics

**DOI:** 10.1002/cns.70854

**Published:** 2026-06-11

**Authors:** Shiyang Weng, Kai Wu, Yinjun Huang, Lei Cao, Huichao Fu

**Affiliations:** ^1^ Department of Trauma Center Shanghai General Hospital, Shanghai Jiao Tong University School of Medicine Shanghai China

**Keywords:** autophagy, ferroptosis, neurology, spinal cord ischemia reperfusion injury

## Abstract

**Objective:**

Spinal cord injury (SCI) is a severe secondary injury that often results in impaired motor function, imposing a significant burden on both individuals and society. Therefore, there is an urgent need for new therapeutic targets and strategies to address this challenge.

**Methods:**

To construct a mouse model of SCI, an aneurysm clip was used in vivo to clamp the abdominal aorta below the left renal artery in C57BL/6J mice. After 60 min, the aneurysm clip was removed to restore blood flow. In vitro, primary neuronal cells were subjected to OGD/R to mimic the conditions of SCI. Cell viability was assessed using the CCK‐8 assay, and the levels of neuronal death, autophagy, and ferroptosis were determined using a combination of WB, IF, and transmission electron microscopy. IP/MS and Co‐IP techniques were employed for the identification and validation of proteins interacting with USP5.

**Results:**

Neurons exhibit significant ferroptosis in the SCI mice. USP5 is markedly upregulated in SCI neurons and mediates neural ferroptosis. In vitro experiments demonstrate that overexpression of USP5 promotes neuronal ferroptosis, whereas knockout of USP5 significantly reduces it, with consistent results observed in vivo. Notably, the upregulation of USP5 expression markedly increases the accumulation of autophagosomes and autophagic flux in neurons, which may represent a potential mechanism by which USP5 mediates neuronal ferroptosis. Further investigations utilizing IP/MS and Co‐IP confirmed the interaction between USP5 and c‐MAF. Additionally, Western blot analysis revealed that USP5, through its deubiquitinating enzyme activity, enhances c‐MAF protein stability, thereby activating autophagy and subsequently promoting neuronal ferroptosis.

**Conclusion:**

In summary, our results indicate a close relationship between ferroptosis and SCI. USP5 regulates c‐MAF expression through deubiquitination, thereby activating autophagy‐dependent ferroptosis in neurons and mediating the progression of SCI. USP5 may serve as a potential therapeutic target for SCI.

## Graphical abstract

SCI induces significant upregulation of USP5 in neurons. The overexpressed USP5 stabilizes c‐MAF protein expression through its deubiquitinase activity, activating autophagy and accelerating the degradation of autophagic ferritin, thereby mediating neuronal ferroptosis. This ultimately leads to impaired functional recovery in SCI mice.

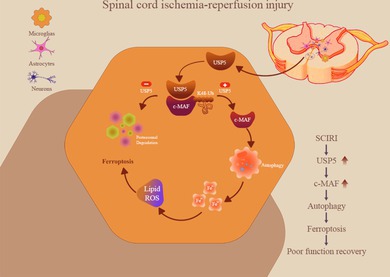

## 1 Introduction

Spinal cord ischemia–reperfusion injury (SCI) is a severe neurological condition that arises from the transient interruption of spinal cord blood flow, followed by the restoration of circulation [1, 2]. The reperfusion phase initiates a cascade of complex cellular and molecular events, including excessive oxidative stress, the generation of reactive oxygen species, activation of local and systemic inflammatory responses, and widespread neuronal apoptosis or necrosis [3, 4]. Neuronal survival is a critical determinant of spinal cord recovery. It has been shown that neurons contribute to repair by upregulating neurotrophic factors such as nerve growth factor (NGF) and brain‐derived neurotrophic factor (BDNF), facilitating synaptic remodeling and circuit reorganization, and ultimately supporting the restoration of neural function [5, 6]. However, extensive neuronal damage may lead to a decreased regenerative capacity, limiting the potential for repair [7, 8]. Thus, preserving neuronal integrity during SCI is essential for improving functional outcomes and accelerating patient recovery.

Ferroptosis is a regulated form of cell death characterized by iron‐dependent lipid peroxidation and is mechanistically driven by inactivation of glutathione peroxidase 4 (GPX4), iron overload, and impaired antioxidant defenses [9, 10]. While originally studied in cancer and neurodegenerative diseases [11, 12], ferroptosis has recently emerged as a potential contributor to central nervous system injuries [13], including SCI, though its precise role and upstream regulatory mechanisms remain largely undefined. Autophagy, a fundamental cellular recycling process, plays a dual role in this context. On the one hand, autophagy helps maintain cellular homeostasis by degrading damaged organelles and proteins [14, 15]; on the other hand, it facilitates ferroptosis by degrading ferritin to release free iron and promoting lipid peroxidation [16, 17]. This dynamic and context‐dependent crosstalk between autophagy and ferroptosis is increasingly recognized as a critical modulator of cell fate under stress conditions. Understanding this interplay may offer new avenues for therapeutic intervention in SCI and other neuroinflammatory disorders.

Ubiquitination and deubiquitination are critical post‐translational modifications that regulate protein homeostasis and cell fate. Recent studies have shown that TRIM56 mitigates neuronal PANoptosis in SCI by promoting the ubiquitin‐mediated degradation of YBX1 [18], highlighting the pivotal role of the ubiquitin system in SCI. As a major deubiquitinating enzyme, USP5 is involved in multiple physiological processes, including DNA repair and the regulation of cell death. Previous reports have demonstrated that USP5 suppresses ferroptosis in breast cancer by deubiquitinating GPX4 [19] and regulates ferroptosis in colorectal cancer by promoting lysosome‐dependent degradation of the YBX3/SLC7A11 axis [20], highlighting its potential role in both ferroptosis and autophagy‐related pathways. However, the function of USP5 in neuronal survival and SCI has yet to be fully elucidated.

Therefore, we hypothesize that USP5 promotes SCI progression by inducing autophagy‐dependent ferroptosis in neurons. Targeting USP5 may thus represent a promising therapeutic strategy to mitigate neuronal injury following spinal cord ischemia–reperfusion.

## 2 Methods

### 2.1 Isolation and Culture of Primary Neurons

The extraction of neurons was performed as previously described [21]. The purity of the neurons was confirmed by immunofluorescence staining using neuron‐specific markers. The isolated neurons were seeded on poly‐D‐lysine‐coated plates and cultured under specified conditions.

### 2.2 Adenovirus Transfection

Neurons were transfected with adenoviral constructs at DIV 7 using the following groups: Adenoviral shRNA control (Ad‐shCtrl), adenoviral shRNA targeting ATG5 (Ad‐shATG5), USP5 (Ad‐shUSP5), c‐MAF (Ad‐shc‐MAF), adenoviral vector control (Ad‐Vec), and adenoviral expression constructs for USP5 (Ad‐USP5) or c‐MAF (Ad‐c‐MAF). After a 6‐h incubation with the adenoviral particles, the medium was replaced with fresh Neurobasal complete medium. Transfection efficiency and knockdown or overexpression of target genes were confirmed 48 h post‐infection using fluorescence microscopy and quantitative PCR.

### 2.3 Animal

USP5^−/−^ mice were generated using the CRISPR/Cas9 gene‐editing technology. Specifically, two single guide RNAs (sgRNAs) targeting exon 3 of the mouse USP5 gene were designed to induce double‐strand breaks. The sgRNAs were synthesized and cloned into the px330 vector, which contains the Cas9 endonuclease. The recombinant plasmids were then co‐injected into fertilized C57BL/6 mouse zygotes along with in vitro transcribed Cas9 mRNA. Injected embryos were implanted into pseudopregnant foster mothers. Genomic DNA from tail biopsies of offspring was analyzed by PCR and sequencing to identify deletions or insertions at the target site. Homozygous knockout mice were confirmed by Western blotting for the absence of USP5 protein. Knockout mice and wild‐type littermates were maintained in a specific pathogen‐free facility for subsequent experiments. The animal experiment was approved by the Ethics Committee of Shanghai General Hospital.

### 2.4 SCI Mouse Model

All procedures involving animals were conducted in accordance with the National Institutes of Health Guide for the Care and Use of Laboratory Animals (approval number: 2025AW229). Mice were housed in a temperature‐controlled (22° ± 2°C) and humidity‐controlled (50% ± 10%) environment with a 12 h light/dark cycle, with ad libitum access to food and water. After at least one week of acclimatization, an SCI model was established at the T10 vertebral level using a modified Allen's impactor. Briefly, mice were anesthetized using −3% isoflurane delivered in 100% oxygen via a nose cone and maintained at 1%–2% isoflurane throughout the surgical procedure. A midline dorsal incision was made, and the paravertebral muscles were separated to expose the T10 vertebra. A laminectomy was performed to expose the dorsal surface of the spinal cord without damaging the dura. A moderate contusion injury was induced by dropping a 10 g rod from a height of 20 mm onto the exposed spinal cord. Successful modeling was confirmed by immediate hemorrhage and edema, dura swelling, and characteristic tail and hind limb responses. Four weeks after injury, mice were euthanized via gradually increasing CO_2_ inhalation until respiratory and cardiac arrest were confirmed. Spinal cord tissues were collected, fixed in 4% paraformaldehyde, paraffin‐embedded, and subjected to H&E staining for histological analysis.

### 2.5 Neuronal Treatment With OGD/R

Primary neurons were subjected to oxygen–glucose deprivation and reoxygenation (OGD/R) to simulate ischemia–reperfusion injury in vitro. Neurons cultured for 7–10 days in vitro (DIV7‐10) were transferred to a glucose‐free medium and placed in a hypoxic chamber (1% O_2_, 5% CO_2_, 94% N_2_) at 37°C for 2 h to induce OGD. After the deprivation period, the neurons were returned to a normoxic environment, and the original culture medium with glucose was replaced. The cells were then incubated under normal conditions (95% air, 5% CO_2_) for the designated reoxygenation time. The treated neurons were subsequently analyzed for various assays.

### 2.6 Western Blotting (WB)

Cells or tissue samples were lysed in RIPA buffer containing protease and phosphatase inhibitors. Protein concentrations were determined using the BCA assay (A55866, ThermoFisher Scientific). Equal amounts of protein were separated by SDS‐PAGE and transferred onto PVDF membranes. Membranes were blocked with 5% non‐fat milk in TBST (TBS with 0.1% Tween‐20) for 1 h at room temperature. They were then incubated overnight at 4°C with primary antibodies specific to the target proteins. After washing, membranes were incubated with HRP‐conjugated secondary antibodies for 1 h at room temperature. The protein bands were visualized using an ECL detection system and imaged. Quantification was performed using ImageJ. The specific information of the primary antibody is provided in Table S1.

### 2.7 Immunofluorescence (IF)

Cells were fixed with 4% paraformaldehyde (PFA) for 20 min at room temperature. After washing with PBS, cells were permeabilized with 0.4% Triton X‐100 in PBS for 10 min. Blocking was performed with 5% BSA in PBS for 1 h at room temperature. The cells were incubated overnight at 4°C with primary antibodies diluted in blocking buffer. After washing with PBS, cells were incubated with fluorescently labeled secondary antibodies for 1 h at room temperature in the dark. Nuclei were counterstained with DAPI, then imaged using a laser confocal microscope (Olympus FV3000, Tokyo, Japan).

### 2.8 GSH, 4‐HNE, MDA, Fe^2+^ Level Detection

Glutathione (GSH) assay kit (A006‐1‐1, Jiancheng, China), 4‐Hydroxynonenal ELISA kit (H268‐1‐1, Jiancheng, China), Malondialdehyde (MDA) assay kit (A003‐1‐2, Jiancheng, China), and iron Assay Kit (ab83366, Abcam) were used to detect the levels of GSH, 4‐HNE, MDA, and Fe^2+^ in cells and tissues.

### 2.9 Single‐Cell Analysis

The GSE162610 dataset was obtained from GEO. Quality control was conducted to filter out low‐quality cells and genes with low expression. Subsequent data analysis included dimensionality reduction using t‐SNE, clustering of cells into distinct populations, and differential gene expression analysis to identify markers for each cell cluster.

### 2.10 Detection of ROS Levels

ROS levels in spinal cord tissues were measured using a DCFH‐DA assay kit (S0033S, Beyotime) following the manufacturer's instructions. Briefly, after treatment, cells were incubated with 10 μM DCFH‐DA for 20 min at 37°C in the dark, then washed with PBS. Fluorescence intensity was measured using a microplate reader. For spinal cord tissues, homogenates were incubated with 10 μM DCFH‐DA at 37°C for 30 min to detect ROS. After incubation, the samples were washed with PBS, and ROS levels were assessed by measuring the fluorescence intensity of DCF (excitation: 485 nm, emission: 530 nm) using a fluorescence microplate reader.

On the other hand, C11‐BODIPY 581/591 is a fluorescent probe used to detect lipid peroxidation. Neuronal cells were incubated with the oxidative stress indicator C11‐BODIPY 581/591 (#D3861, Thermo Fisher) according to the manufacturer's instructions. Cells were incubated at 37°C for 30 min, then washed three times with PBS. Fluorescence microscope images were taken to record green and red fluorescence signals. Green fluorescence indicated lipid peroxidation, while red fluorescence indicated non‐peroxidized cells. Finally, the intensity of the observed green fluorescence was quantified and analyzed using ImageJ software.

### 2.11 Electromyography Analysis

To assess neuromuscular function in the SCI mouse model, electromyography (EMG) was performed [22]. Mice were anesthetized with isoflurane and positioned on a heated platform. A pair of needle electrodes was inserted into the gastrocnemius muscle, and a reference electrode was placed subcutaneously near the base of the tail. Electrical stimuli were delivered to the sciatic nerve via a stimulator, and compound muscle action potentials (CMAPs) were recorded. The amplitude and latency of CMAPs were measured using an EMG recording system. The data were analyzed to evaluate the degree of functional recovery following SCI and various experimental treatments.

### 2.12 H&E and Nissl Staining

HE staining and Nissl staining of mouse spinal cord tissue were performed according to the previously provided method [23].

### 2.13 Transmission Electron Microscopy (TEM)

Cells were fixed in 2.5% glutaraldehyde in 0.1 M phosphate buffer (pH 7.4) at 4°C overnight. The samples were then post‐fixed in 1% osmium tetroxide, dehydrated through a graded ethanol series, and embedded in epoxy resin. Ultrathin sections (70–90 nm) were cut using an ultramicrotome and collected on copper grids. The sections were stained with uranyl acetate and lead citrate. Images were acquired using a transmission electron microscope at an accelerating voltage of 80 kV.

### 2.14 PI Staining

Neuronal cell death was evaluated using Propidium Iodide (PI) staining. Cultured neurons were incubated with PI (5 μg/mL, HY‐D0815, MedChemExpress) at 37°C for 10 min. After incubation, the cells were washed with PBS to remove excess dye. PI‐positive (dead) cells were visualized under a fluorescence microscope, and the fluorescence intensity was quantified to determine the percentage of cell death.

### 2.15 Co‐Immunoprecipitation (Co‐IP)

Cells were lysed in a RIPA buffer (20 mM Tris–HCl pH 7.5, 150 mM NaCl, 1% NP‐40, 0.1% SDS, 0.5% sodium deoxycholate) supplemented with protease inhibitors. Lysates were cleared by centrifugation at 12,000 × g for 15 min at 4°C. Supernatants were incubated with specific antibodies or control IgG overnight at 4°C, followed by incubation with Protein A/G agarose beads for 2 h at 4°C. The beads were then washed with lysis buffer, and bound proteins were eluted with SDS‐PAGE sample buffer. Eluates were analyzed by Western blotting using appropriate antibodies.

### 2.16 Immunoprecipitation‐Mass Spectrometry (IP‐MS)

IP‐MS analysis was performed using a Q Exactive Plus mass spectrometer (Thermo Scientific) coupled to an EASY‐nLC 1200 LC/MS system (Thermo Scientific).

### 2.17 Statistical Analysis

All experimental data were analyzed using GraphPad Prism software. Statistical comparisons between groups were made using one‐way ANOVA followed by Tukey's post hoc test or two‐tailed Student's *t*‐test, as appropriate. A *p*‐value of less than 0.05 was considered statistically significant.

## 3 Results

### 3.1 USP5 Regulates Neuronal Ferroptosis Following SCI

To determine whether neurons undergo ferroptosis during spinal cord ischemia–reperfusion, we treated primary neurons with OGD/R or the ferroptosis inducer Erastin. Both treatments significantly reduced neuronal viability (*p* < 0.001), accompanied by decreased GSH and increased MDA, 4‐HNE, Fe^2+^, and ROS levels (*p* < 0.001) (Figure S1A–D). Western blot analysis revealed upregulation of ACSL4 and downregulation of GPX4 and FTH1 (*p* < 0.01), consistent with ferroptosis changes (Figure S1F,G). Notably, these alterations were reversed by the ferroptosis inhibitor Liproxstatin‐1 (*p* < 0.001, Figure S1H–K).

**FIGURE 1:**
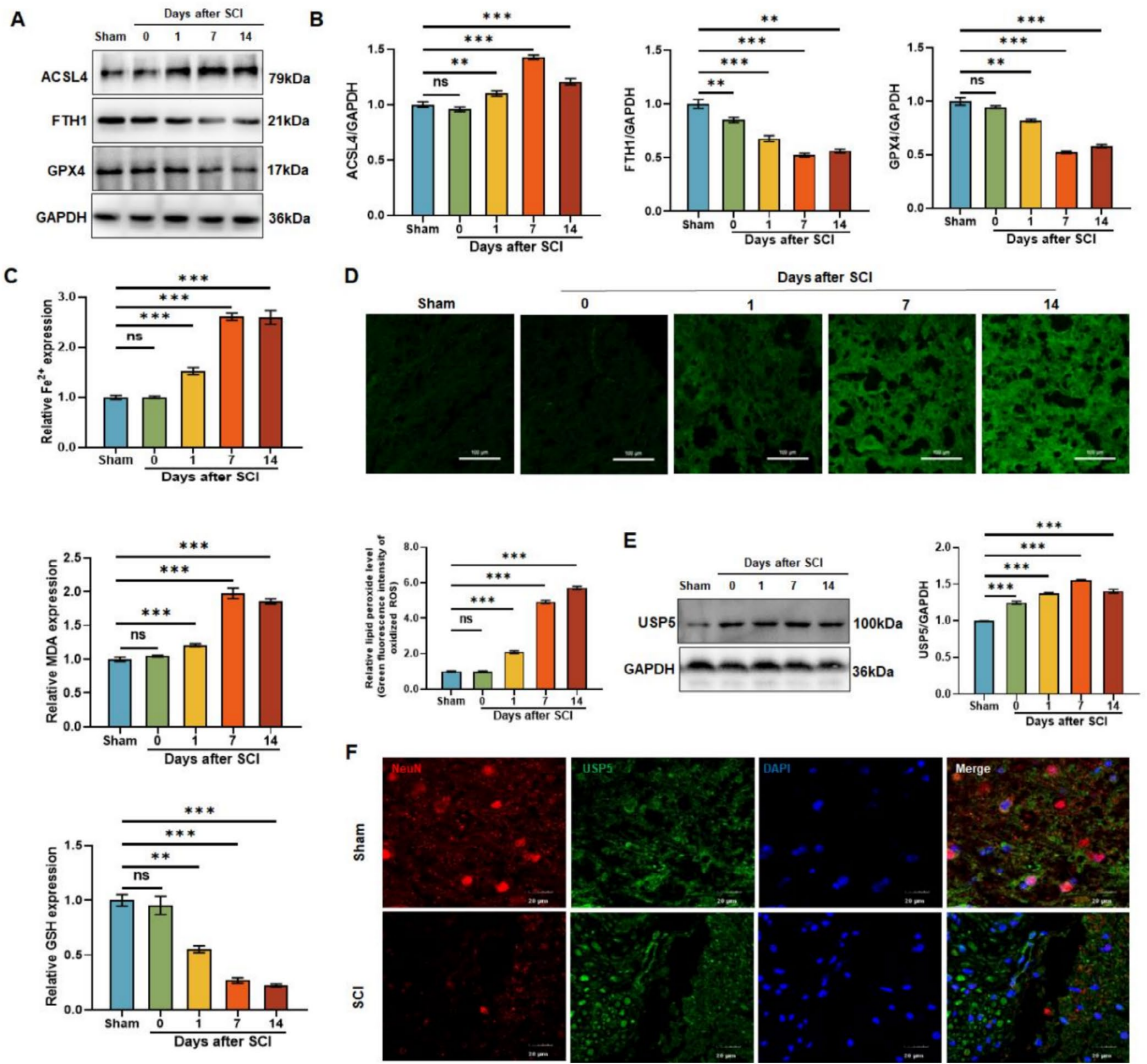
Ferroptosis in neurons of SCI mice. (A) Representative Western blot analysis showing the expression of ferroptosis‐associated proteins ACSL4, FTH1, and GPX4 in spinal cord tissues at the indicated time points (0, 1, 7, and 14 days) after SCI, with sham‐operated mice as controls. GAPDH was used as a loading control. (B) Quantification of protein band intensities from panel A, normalized to GAPDH. (C) Measurement of glutathione (GSH), ferrous iron (Fe^2+^), and malondialdehyde (MDA) levels in spinal cord tissues from sham and I/R‐injured mice at different time points. (D) Representative images of ROS staining in spinal cord sections from each group, with quantification of the corrected total fluorescence intensity (CTFI) of DCF‐positive cells on the right. Scale bars, 100 μm. (E) Western blot analysis and quantification of USP5 protein expression in spinal cord tissues at various time points after SCI. (F) Representative immunofluorescence staining showing co‐localization of USP5 (green) and the neuronal marker NeuN (red) in spinal cord sections from sham and SCI groups. Nuclei were counterstained with DAPI (blue). Scale bars, 20 μm. The data are presented as the mean ± SEM (*n* = 3). ns, *p* > 0.05, **p < 0.01, and ***p < 0.001.

In vivo, mice subjected to SCI exhibited elevated ACSL4, GPX4, and FTH1 protein levels in spinal cord tissues, along with reduced GSH and increased MDA, Fe^2+^, and ROS levels compared to sham controls (*p* < 0.001, Figure 1A–D). Liproxstatin‐1 administration alleviated these ferroptosis‐associated changes (Figure S2), confirming the involvement of ferroptosis in SCI pathology.

Moreover, both Western blot and immunofluorescence analyses demonstrated that USP5 expression was significantly upregulated in SCI spinal cords and OGD/R‐treated neurons (*p* < 0.01, Figure 1E,F, and Figure S3 A,B). Single‐cell transcriptomic analysis, together with immunofluorescence co‐localization, revealed that USP5 is expressed in both neurons and astrocytes, with a prominent enrichment in neurons (Figure S3 C,D). Collectively, these findings indicate that USP5 participates in SCI progression, at least in part, by facilitating neuronal ferroptosis.

### 3.2 High Expression of USP5 as a Critical Mediator of Neuronal Ferroptosis

**FIGURE 2:**
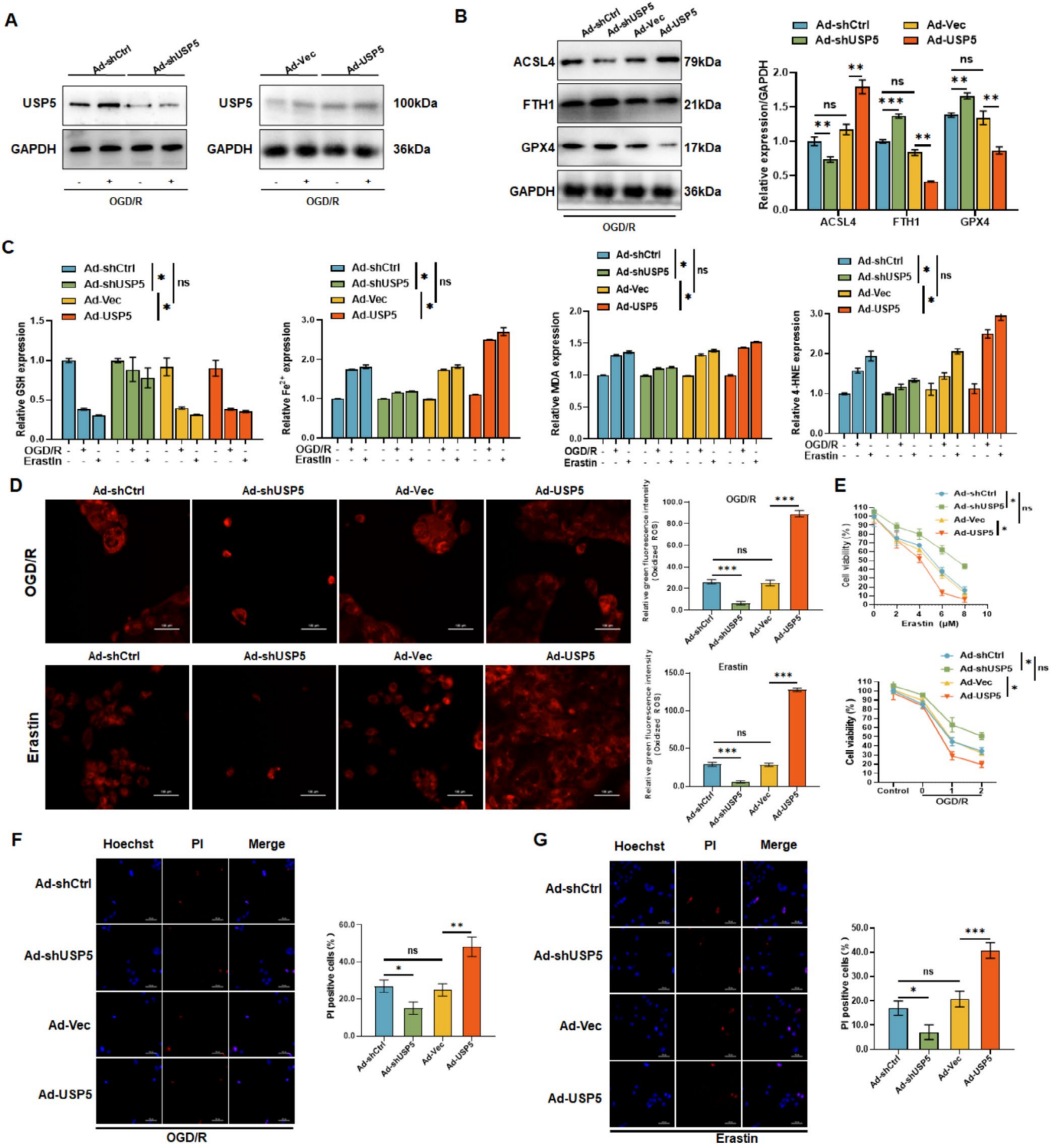
High USP5 expression promotes ferroptosis in neurons. (A) Western blot analysis of USP5 protein expression in primary neurons infected with adenoviruses encoding USP5 (Ad‐USP5), USP5 shRNA (Ad‐shUSP5), or control vectors (Ad‐shCtrl, Ad‐Vec), with or without oxygen–glucose deprivation/reoxygenation (OGD/R) treatment. GAPDH served as a loading control. (B) WB analysis of ferroptosis‐associated proteins ACSL4, FTH1, and GPX4 in the same neuronal groups under OGD/R, with corresponding quantification on the right. € Quantification of GSH, MDA, 4‐hydroxynonenal (4‐HNE), and Fe^2+^ levels in neurons overexpressing or silencing USP5 under either OGD/R or Erastin treatment. (D) Representative images of ROS staining in neurons under OGD/R (top) or Erastin treatment (bottom). Corrected total fluorescence intensity (CTFI) of ROS‐positive cells is shown in the corresponding bar graphs. Scale bars, 100 μm. (E) Cell viability of different neuronal groups exposed to a range of Erastin concentrations (top) or OGD/R (bottom), as measured by CCK‐8 assay. (F, G) Representative images of PI (propidium iodide) staining in neurons under OGD/R (F) or Erastin (G) treatment, with Hoechst counterstaining of nuclei (blue). Quantification of PI‐positive cells is shown in the bar graphs. Scale bars, 100 μm. The data are presented as the mean ± SEm. ns, *p* > 0.05, *p < 0.05, **p < 0.01, and ***p < 0.001.

To investigate the role of USP5 in neuronal ferroptosis, we constructed USP5‐overexpressing and USP5‐silenced neurons (Figure 2A). Western blot analysis revealed that USP5 overexpression significantly increased ACSL4 levels while decreasing FTH1 and GPX4 expression (*p* < 0.01, Figure 2B). This was accompanied by reduced GSH levels and elevated Fe^2+^, MDA, and 4‐HNE concentrations (*p* < 0.05, Figure 2C). Conversely, USP5 knockdown reversed these ferroptosis‐associated changes. Additionally, USP5 overexpression significantly increased ROS levels (*p* < 0.001), decreased cell viability (*p* < 0.05), and promoted neuronal death (*p* < 0.001, Figure 2D–G), whereas USP5 knockdown conferred protective effects.

**FIGURE 3:**
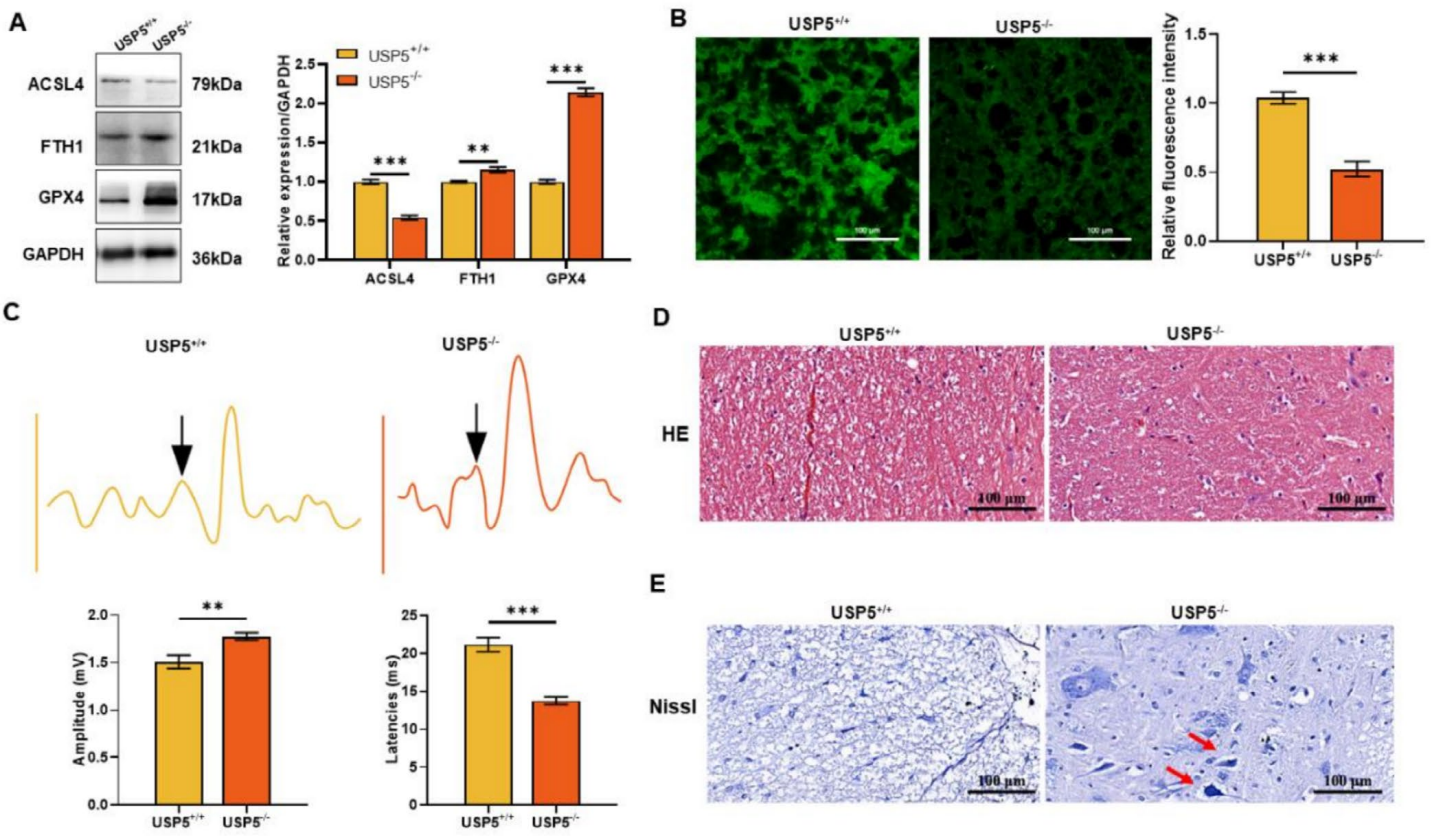
Inhibition of USP5 suppresses ferroptosis and promotes functional recovery in SCIRI mice. (A) Representative Western blot images and quantitative analysis of ferroptosis‐associated proteins ACSL4, FTH1, and GPX4 in spinal cord tissues of wild‐type (USP5^+/+^) and USP5 knockout (USP5^−/−^) mice. GAPDH was used as a loading control. (B) ROS levels detected by fluorescence staining in spinal cord tissues of USP5^+/+^ and USP5^−/−^ mice. Quantification of fluorescence intensity is shown on the right. Scale bars, 100 μm. (C) Electrophysiological assessment of neural function using electromyography (EMG) in USP5^+/+^ and USP5^−/−^ mice. Representative waveforms are shown (top), with quantification of amplitude and latency (bottom). (D) HE staining of spinal cord sections from USP5^+/+^ and USP5^−/−^ mice. Scale bars, 100 μm. (E) Nissl staining showing the morphology and density of neurons in spinal cord tissues. Red arrows indicate preserved Nissl bodies in USP5^−/−^ mice. Scale bars, 100 μm. The data are presented as the mean ± SEM (*n* = 3). *p < 0.05, **p < 0.01, and ***p < 0.001.

**FIGURE 4:**
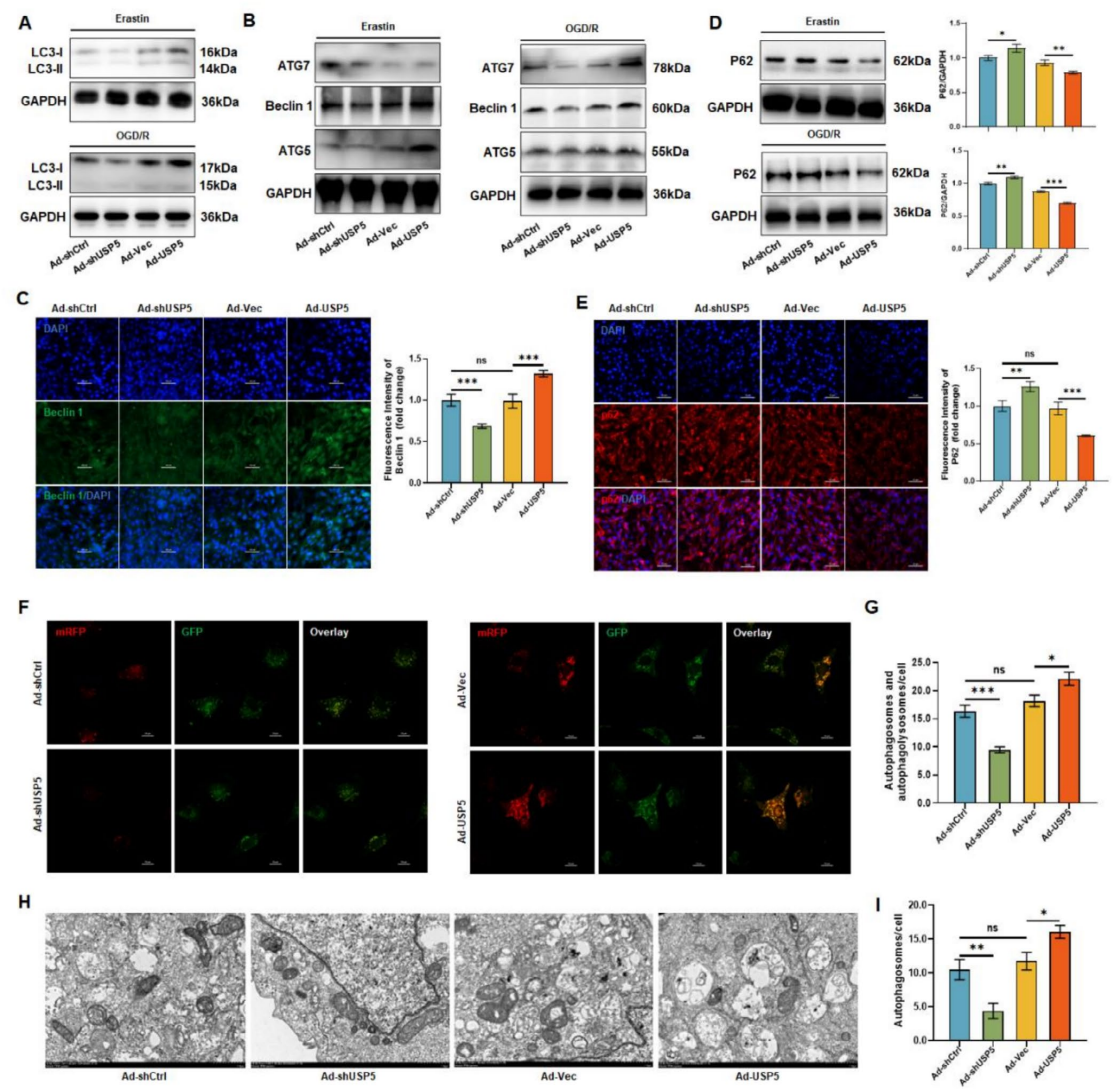
USP5 regulates neuronal autophagy. (A, B) Western blot (WB) analysis showing the expression of autophagy‐related markers LC3‐I/II, ATG7, Beclin‐1, and ATG5 in neurons infected with Ad‐shUSP5, Ad‐USP5, or respective controls (Ad‐shCtrl, Ad‐Vec) under OGD/R or Erastin treatment. GAPDH was used as the loading control. (C) Representative IF images of Beclin‐1 in neurons under the indicated treatments. Nuclei were counterstained with DAPI (blue). Quantification of Beclin‐1 fluorescence intensity is shown on the right. Scale bars, 100 μm. (D) WB images and quantitative analysis of autophagy substrate p62 in neurons with altered USP5 expression under OGD/R or Erastin exposure. (E) IF images of p62 accumulation (red) and DAPI (blue) staining in neurons with USP5 knockdown or overexpression. Scale bars, 100 μm. (F) Fluorescence microscopy images of neurons transduced with tandem mRFP‐GFP‐LC3 adenovirus showing autophagosome (yellow puncta) and autolysosome (red‐only puncta) formation under different USP5 conditions. Scale bars, 10 μm. (G) Quantification of autophagic structures per cell from panel F. (H, I) Transmission electron microscopy (TEM) analysis of neuronal autophagosome structures. Representative TEM images (H) and quantification of autophagosomes per cell (I) are shown. The data are presented as the mean ± SEM. ns, *p* > 0.05, *p < 0.05, **p < 0.01, and ***p < 0.001.

To further assess the in vivo relevance of USP5, we generated USP5 knockout (USP5^−/−^) mice. Successful gene deletion in spinal cord tissues and neurons was confirmed by Western blotting (Figure S4 A,B), with no detectable abnormalities in spinal cord development or baseline neuronal numbers between USP5^−/−^ and wild‐type (WT) mice (Figure S4 C–E). Upon SCI induction, USP5^−/−^ mice exhibited significantly reduced ferroptosis in spinal cord tissues, evidenced by downregulated ACSL4 and upregulated GPX4 and FTH1 expression (*p* < 0.001, Figure 3A). ROS levels, as shown by DHE staining, were also markedly decreased in USP5‐deficient mice compared to WT (*p* < 0.001, Figure 3B). Electromyography analysis demonstrated improved neurological function in USP5^−/−^ mice, with higher motor‐evoked potential (MEP) amplitudes and shorter latencies (*p* < 0.001, Figure 3C). Histological analyses (H&E and Nissl staining) further confirmed that USP5 deletion mitigated neuronal loss, vacuolization, necrosis, and vascular damage post‐SCI (Figure 4D,E).

Moreover, overexpression of USP5 in SCI mice exacerbated ferroptosis and neuronal damage, as indicated by increased ACSL4, decreased GPX4/FTH1, elevated ROS, and worsened histopathological outcomes (*p* < 0.01, Figure S5). Together, these findings suggest that high USP5 expression promotes neuronal ferroptosis and impairs neurological recovery following SCI.

### 3.3 USP5 Regulates Neuronal Autophagy‐Dependent Ferroptosis

Given the close interplay between autophagy and ferroptosis, we next investigated whether USP5 regulates neuronal autophagy. Western blot analysis revealed that USP5 overexpression significantly increased LC3‐II conversion and upregulated the expression of ATG7, ATG5, and Beclin‐1, while USP5 knockdown had the opposite effect (*p* < 0.001, Figure 4A–C). The autophagy substrate p62 was also markedly elevated upon USP5 overexpression and reduced after USP5 silencing (*p* < 0.05, Figure 4D,E). mRFP‐GFP‐LC3 reporter assays confirmed that USP5 significantly enhanced autophagic flux in neurons, whereas USP5 depletion impaired autophagic flux (*p* < 0.01, Figure 4F,G). Consistently, transmission electron microscopy (TEM) showed that USP5 deficiency decreased the number of autophagosomes and autolysosomes, while USP5 overexpression promoted their accumulation (*p* < 0.05, Figure 4H,I).

**FIGURE 5:**
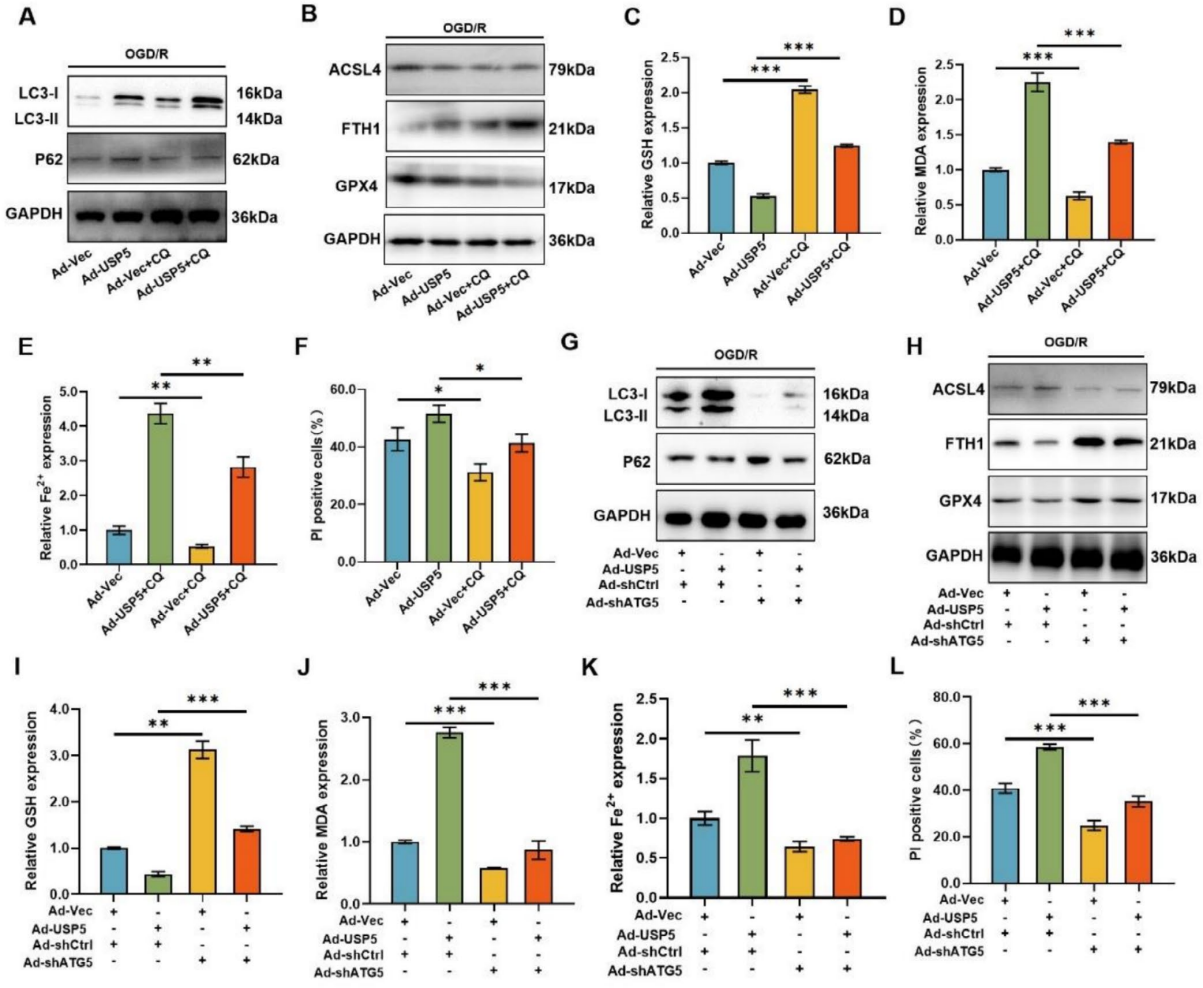
Inhibition of autophagy attenuates neuronal ferroptosis. (A, B) Western blot analysis of autophagy markers (LC3‐I/II and p62) and ferroptosis‐related proteins (ACSL4, FTH1, GPX4) in neurons overexpressing USP5 with or without autophagy blockade using chloroquine (CQ, 10 μM). (C–E) Quantification of GSH, MDA, and Fe^2+^ levels in neurons under the indicated treatments. (F) Flow cytometric analysis of PI‐positive cells in neurons overexpressing USP5, with or without CQ treatment. (G, H) WB analysis of LC3‐I/II, p62, ACSL4, FTH1, and GPX4 expression in neurons overexpressing USP5 with or without co‐infection of Ad‐shATG5. (I–K) Biochemical assessment of intracellular GSH, MDA, and Fe^2+^ levels in neurons overexpressing USP5, with or without ATG5 knockdown. (L) Percentage of PI‐positive cells in neurons treated as indicated, assessed by flow cytometry. The data are presented as the mean ± SEM. *p < 0.05, **p < 0.01, and ***p < 0.001.

To further assess whether USP5‐induced ferroptosis is autophagy‐dependent, we inhibited autophagy using chloroquine (CQ) or Ad‐shATG5 (Figure 5A,G). Both pharmacological and genetic inhibition of autophagy reversed the ferroptosis‐related protein changes induced by USP5 overexpression, including upregulation of ACSL4 and downregulation of GPX4 and FTH1 (Figure 5B,H). Moreover, CQ or Ad‐shATG5 treatment significantly alleviated OGD/R‐induced ferroptotic changes, such as reduced GSH and elevated MDA and Fe^2+^ levels (*p* < 0.01, Figure 5C–E, 5I–K). In addition, the OGD/R‐induced decrease in neuronal viability and increase in cell death were significantly rescued by autophagy inhibition (*p* < 0.01, Figure 5F,L). Collectively, these findings demonstrate that USP5 promotes autophagy activation, which is required for ferroptosis execution in neurons.

### 3.4 USP5‐Mediated Deubiquitination Regulates c‐MAF

**FIGURE 6:**
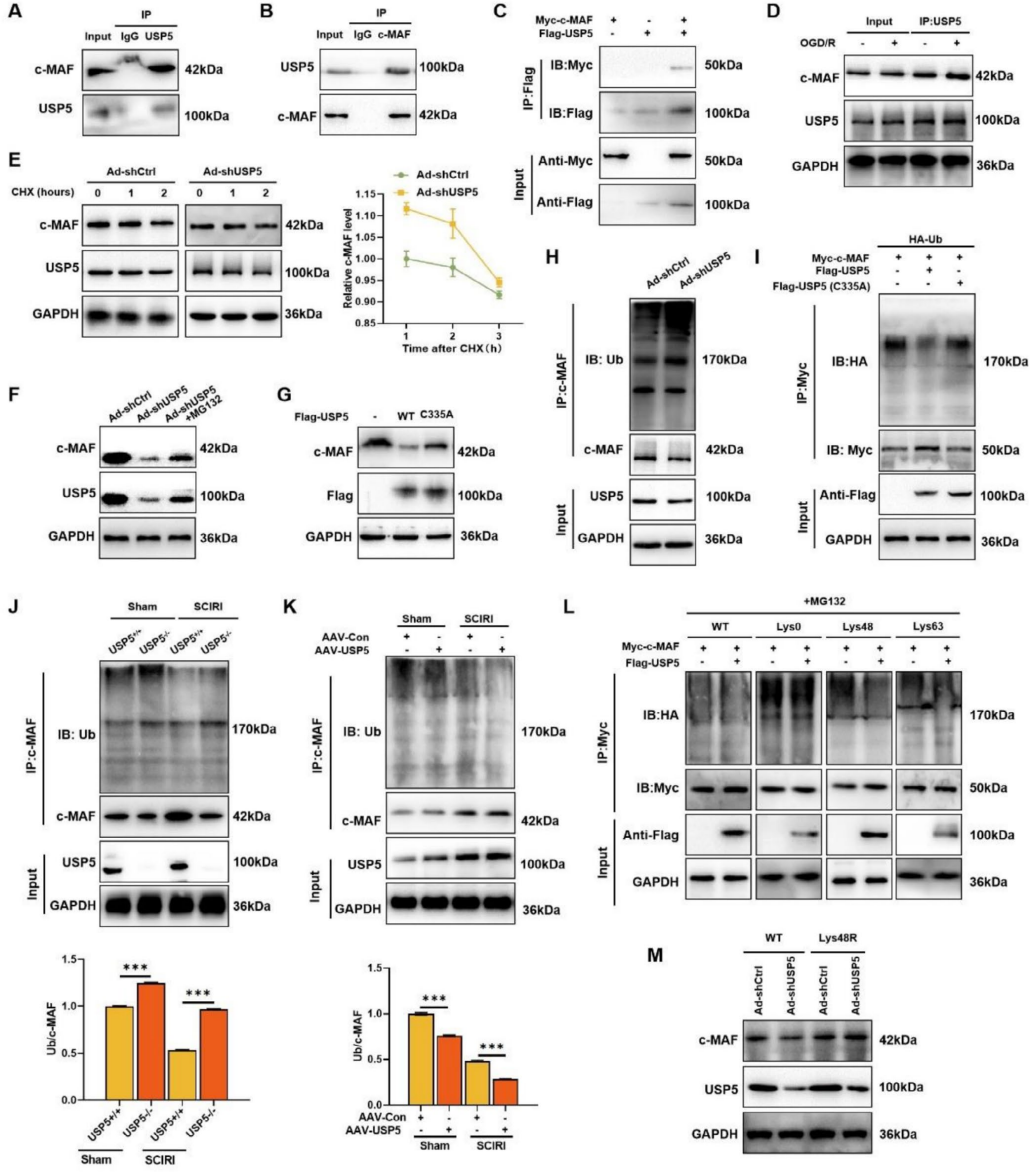
USP5 ubiquitination regulates c‐MAF protein stability. (A, B) Co‐immunoprecipitation (Co‐IP) assays in neurons and HEK293T cells confirm the endogenous interaction between USP5 and c‐MAF. (C) Exogenous Co‐IP validation of USP5–c‐MAF interaction in HEK293T cells co‐transfected with Myc‐c‐MAF and Flag‐USP5. (D) Co‐IP showing enhanced USP5–c‐MAF interaction under OGD/R stimulation in neurons. (E) Cycloheximide (CHX, 10 μg/mL) chase assay shows that USP5 knockdown reduces c‐MAF protein stability over time (0–3 h). (F) MG132 treatment (10 μM, 6 h) partially restores c‐MAF expression in USP5‐silenced neurons, suggesting proteasome‐dependent degradation. (G) Western blot showing that the deubiquitinating‐inactive mutant USP5‐C335A fails to stabilize c‐MAF compared with WT‐USP5. (H, I) Ubiquitination assays showing increased c‐MAF ubiquitination in USP5‐silenced neurons and in HEK293T cells co‐transfected with HA‐Ub and WT or mutant USP5 constructs. (J, K) In vivo analysis of c‐MAF ubiquitination levels in spinal cord tissues of USP5^+/+^ vs. USP5^−/−^ mice (J) and in AAV‐USP5 vs. AAV‐Control treated mice (K). (L) Ubiquitination assay reveals USP5 specifically removes K48‐linked, but not K63‐linked, ubiquitin chains from c‐MAF. (M) Western blot analysis of c‐MAF levels in USP5‐knockdown neurons transfected with WT or ubiquitination‐defective K48R constructs, confirming USP5‐mediated stabilization of c‐MAF via K48 deubiquitination. The data are presented as the mean ± SEM. **p < 0.01, ***p < 0.001.

To investigate how USP5 modulates autophagy‐dependent ferroptosis, we performed IP/MS analysis to identify its interacting partners. Both endogenous and exogenous Co‐IP confirmed a robust interaction between USP5 and c‐MAF, which was further enhanced upon OGD/R stimulation (Figure 6A–D). Notably, OGD/R or Erastin treatment increased c‐MAF protein levels, whereas USP5 knockdown significantly reversed this effect (*p* < 0.01, Figure S6), suggesting that USP5 regulates c‐MAF stability.

As a deubiquitinating enzyme, USP5 likely controls c‐MAF expression via ubiquitin‐dependent proteasomal degradation. Cycloheximide (CHX) chase assays demonstrated that USP5 depletion significantly shortened the half‐life of c‐MAF (*p* < 0.05, Figure 6E). Pretreatment with the proteasome inhibitor MG132 restored c‐MAF levels in USP5‐deficient neurons, confirming proteasomal degradation (Figure 6F). Moreover, overexpression of a catalytically inactive USP5 mutant (C335A) markedly reduced c‐MAF stability (Figure 6G).

Ubiquitination assays revealed that USP5 knockdown increased c‐MAF ubiquitination and degradation, while enzymatic inactivation of USP5 produced similar effects (Figure 6H,I). Consistent findings were observed in spinal cord tissues from USP5‐deficient mice, which showed elevated c‐MAF ubiquitination and reduced protein levels, whereas USP5 overexpression stabilized c‐MAF (*p* < 0.001, Figure 6J,K).

We next examined the specificity of USP5 toward polyubiquitin linkages. USP5 selectively removed K48‐linked ubiquitin chains from c‐MAF, without affecting K63‐linked ubiquitination (Figure 6L). Mutation of the K48 site (K48R) abolished USP5‐mediated deubiquitination of c‐MAF (Figure 6M). These data indicate that USP5 stabilizes c‐MAF by removing K48‐linked polyubiquitin chains and preventing its proteasomal degradation.

### 3.5 Inhibition of c‐MAF Reverses USP5‐Mediated Neuronal Ferroptosis

To clarify the role of c‐MAF in USP5‐mediated ferroptosis, we first observed that c‐MAF knockdown significantly reduced neuronal autophagy activation, whereas c‐MAF overexpression enhanced it (Figure S7 A). In neurons co‐transfected with USP5 and c‐MAF siRNA, c‐MAF silencing effectively reversed the USP5‐induced increase in ROS and suppressed ferroptosis‐related markers, including ACSL4 upregulation and GPX4/FTH1 downregulation (*p* < 0.05, Figure S7 C–G). Conversely, co‐overexpression of USP5 and c‐MAF further aggravated ferroptosis injury, as evidenced by enhanced ROS accumulation and ferroptosis marker expression. Cell viability assays showed that c‐MAF knockdown significantly restored USP5‐induced neuronal death, whereas co‐overexpression of USP5 and c‐MAF markedly exacerbated neuronal loss (*p* < 0.05, Figure S7H,I).

**FIGURE 7:**
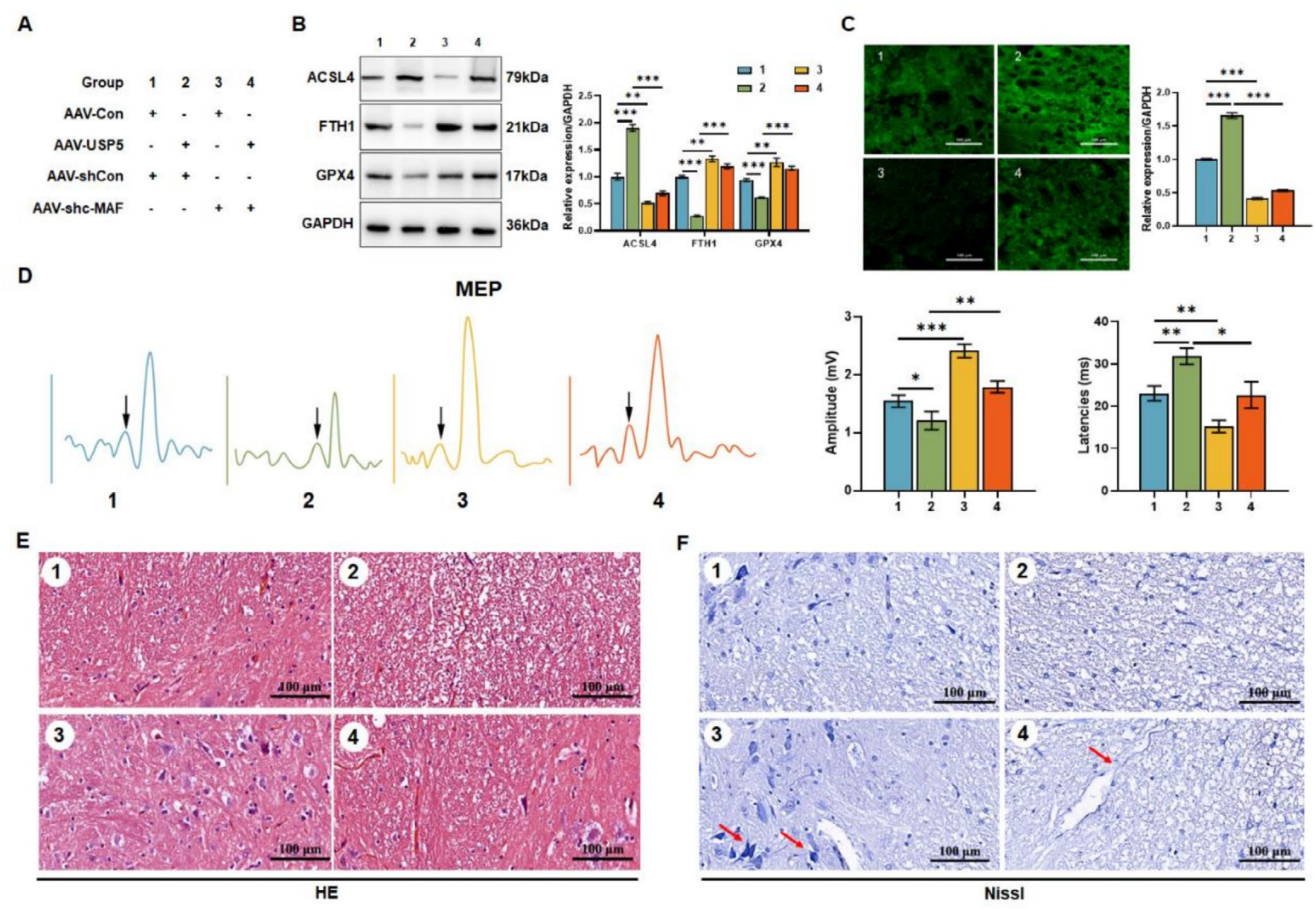
Inhibition of c‐MAF promotes SCIRI functional recovery. (A) Group allocation for animal experiments: Group 1: AAV‐Con; Group 2: AAV‐USP5; Group 3: AAV‐USP5 + AAV‐shCon; Group 4: AAV‐USP5 + AAV‐shc‐MAF. (B) Western blot analysis of ACSL4, FTH1, and GPX4 protein levels in spinal cord tissues. GAPDH was used as the internal control. Quantification was performed using ImageJ. (C) Detection of reactive oxygen species (ROS) levels in spinal cord sections via immunofluorescence staining (green). DAPI was used for nuclear counterstaining. Scale bar: 100 μm. (D) MEP recordings obtained using electromyography. Peak amplitude (mV) and latency (ms) were quantified to evaluate neurophysiological function. (E) HE staining of spinal cord tissue sections. Tissue architecture and cellular morphology were observed under light microscopy. Scale bars: 100 μm. (F) Nissl staining of spinal cord sections for neuronal survival assessment. Nissl bodies were visualized under light microscopy. Scale bar: 100 μm. The data are presented as the mean ± SEM. ns, *p* > 0.05, *p < 0.05, **p < 0.01, and ***p < 0.001.

We then validated these findings in vivo. SCI mice receiving AAV‐mediated USP5 overexpression with c‐MAF knockdown (Figure 7A) showed significant reversal of ACSL4 upregulation and FTH1/GPX4 suppression induced by USP5 overexpression (*p* < 0.001, Figure 7B). DHE staining revealed reduced ROS levels in spinal cord tissues of c‐MAF‐inhibited mice compared to USP5‐overexpressing controls (*p* < 0.001, Figure 7C). Electromyography analysis further demonstrated functional improvement with c‐MAF inhibition, including increased motor‐evoked potential amplitude and reduced latency (*p* < 0.01, Figure 7D).

Histological assessment confirmed that USP5 overexpression led to pronounced neuronal loss, vacuolation, and structural disorganization in SCI spinal cords, all of which were markedly alleviated by c‐MAF inhibition (Figure 7E,F). Together, these findings demonstrate that USP5 promotes neuronal ferroptosis and SCI progression through c‐MAF stabilization and subsequent activation of autophagy‐dependent ferroptosis.

## 4 Discussion

Although numerous studies have elucidated the relationship between ferroptosis and various diseases, the connection between SCI and ferroptosis has yet to be fully clarified, and the specific molecular mechanisms regulating this process remain unknown. In this study, we first demonstrated that ferroptosis occurs in neurons during SCI, with USP5 emerging as a key mediator of this event. USP5 is upregulated in SCI neurons and stabilizes the c‐MAF protein through its deubiquitinase activity, thereby promoting SCI progression via the activation of neuronal autophagy‐dependent ferroptosis.

A key conceptual advance of this study is the identification of USP5 as an upstream amplifier of ferroptosis susceptibility, rather than a passive downstream effector of cell death. DUBs are increasingly recognized as context‐dependent modulators of stress responses [24, 25]; however, their roles in ferroptosis remain incompletely defined. Notably, USP5 has been shown to regulate ferroptosis in a context‐specific manner. For instance, USP5 inhibits ferroptosis in hepatocellular carcinoma by stabilizing LSH, thereby promoting tumor progression [26]. In contrast, a recent study in lung adenocarcinoma (LUAD) reported that TFEB activation promotes TRIM25 binding to GPX4 while competitively disrupting USP5–GPX4 interactions, leading to ferroptosis induction [27]. However, whether USP5 exerts similar or opposing functions in neural tissues under ischemic stress remains unexplored. Our study demonstrates that USP5 is significantly upregulated in neurons following ischemia–reperfusion injury and promotes SCI progression by enhancing neuronal ferroptosis. These findings uncover a previously unrecognized pathological role for USP5 in central nervous system injury and fill a critical gap in our understanding of its function in ferroptosis‐related neurodegeneration.

Autophagy is traditionally viewed as a protective mechanism that maintains cellular homeostasis. For example, enhancing autophagic activity has been shown to reduce neuronal pyroptosis in SCI [28, 29]. However, growing evidence indicates that autophagy can also act as a pro‐death mechanism under certain pathological conditions. In particular, excessive or maladaptive autophagy has been shown to promote ferroptosis by releasing intracellular iron and accelerating lipid peroxidation [30, 31]. In the context of SCI, where iron overload and ROS accumulation are already prominent, USP5‐driven autophagy may lower the threshold for ferroptosis execution. Notably, both pharmacological and genetic inhibition of autophagy mitigated USP5‐induced ferroptosis, supporting a model in which autophagy functions not as a bystander, but as a functional prerequisite for USP5‐mediated neuronal injury.

Interestingly, our single‐cell transcriptomic and immunofluorescence analyses revealed that USP5 is not restricted to neurons but is also expressed in astrocytes. Although the functional consequences of astrocytic USP5 remain unexplored, its presence in both cell types raises the possibility of coordinated neuron–glia interactions that modulate ferroptosis susceptibility. Astrocytes play essential roles in maintaining the extracellular redox balance, iron homeostasis, and glutamate clearance—factors that strongly influence ferroptosis sensitivity [32, 33, 34]. Therefore, USP5 expression in astrocytes may influence neuronal injury indirectly, either by altering the oxidative microenvironment or disrupting metabolic support to neurons. Future studies using cell‐type–specific USP5 ablation will be necessary to delineate the relative contribution of neuronal vs. astrocytic USP5 to SCI pathology.

We further identified c‐MAF as a direct substrate of USP5, adding mechanistic specificity to the ferroptosis axis. Previous studies have primarily focused on the role of c‐MAF in immune and developmental regulation. For instance, c‐MAF has been shown to orchestrate the fate conversion between TFH and TH17 cells in TGF‐β–enriched environments [35] and to protect against pathogen‐induced colitis by regulating immune gene networks [36]. However, its involvement in ferroptosis has remained largely unexplored. Our findings reveal that USP5 stabilizes c‐MAF by removing K48‐linked polyubiquitin chains, suggesting that transcriptional reprogramming may underlie the increased ferroptosis susceptibility induced by USP5. Although c‐MAF did not appear to directly regulate canonical autophagy‐related proteins, it may influence ferroptosis indirectly through transcriptional control of genes involved in redox homeostasis, lipid metabolism, or iron handling. Collectively, these observations suggest that c‐MAF may function as a transcriptional sensitizer, coordinating the convergence of autophagy and ferroptosis pathways under ischemic stress.

From a broader perspective, our findings suggest that neuronal ferroptosis in spinal cord injury is not solely a consequence of metabolic imbalance, but is actively governed by post‐translational regulatory networks. In particular, the USP5–c‐MAF axis may serve as a molecular switch that transforms ischemia‐induced stress into irreversible neuronal loss. This conceptual framework helps explain why ferroptosis is especially prominent during the reperfusion phase—a period characterized by intense ubiquitin‐dependent protein remodeling that may sensitize neurons to ferroptosis.

From a translational perspective, the USP5–c‐MAF axis represents a promising therapeutic target to mitigate ferroptosis‐driven neuronal damage in SCI. Pharmacological inhibition of USP5, or selective disruption of the USP5–c‐MAF interaction, may attenuate autophagy‐dependent ferroptosis and preserve neuronal integrity following injury. Given the increasing recognition of ferroptosis in central nervous system (CNS) pathologies, developing USP5‐selective inhibitors with sufficient blood–spinal cord barrier permeability, as well as evaluating combinatorial strategies involving ferroptosis inhibitors and autophagy modulators in preclinical models, should be prioritized. Ultimately, our findings offer a mechanistic and conceptual foundation for targeting ferroptosis as a clinically actionable vulnerability in spinal cord injury and related neurodegenerative conditions.

## 5 Limitation

Although our study provides compelling evidence that USP5 promotes spinal cord injury progression by stabilizing c‐MAF and activating autophagy‐dependent ferroptosis, several limitations should be acknowledged. First, while we demonstrated the stabilization of c‐MAF by USP5, the downstream transcriptional targets of c‐MAF that mediate ferroptosis sensitivity remain incompletely characterized. Future studies integrating ChIP‐seq and transcriptomic profiling are warranted to uncover these regulatory networks. Second, our in vivo findings were based on a murine SCI model, which may not fully recapitulate the complex pathophysiology of human spinal cord injury. Validation using human spinal cord specimens or iPSC‐derived neuronal organoids could enhance translational relevance. Lastly, the therapeutic potential of pharmacological USP5 inhibition was not addressed in this study. Future investigations into USP5‐targeted compounds and their effects on ferroptosis and neuronal survival in SCI models are necessary to explore potential clinical applications.

## 6 Conclusion

In summary, our results demonstrate that neurons undergo ferroptosis during the SCI process. This study innovatively proposes that USP5 regulates c‐MAF expression through deubiquitination, which activates neuronal autophagy and subsequently promotes neuronal ferroptosis. This contributes to a deeper understanding of the mechanisms underlying ferroptosis in SCI. Additionally, we have shown that knocking out USP5 after SCI exerts a neuroprotective effect. Targeting USP5 may thus represent a potential therapeutic approach for neuronal injury in SCI.

## Author Contributions

Shiyang Weng, Lei Cao, and Huichao Fu conceived, designed, and supervised the whole study; Shiyang Weng, Kai Wu, and Yinjun Huang operated the experiment, performed the analyses, and audited the data; Shiyang Weng and Kai Wu wrote the manuscript; Lei Cao and Huichao Fu revised the manuscript. All authors provided critical comments and approved the final manuscript.

## Funding

The authors have nothing to report.

## Disclosure

The authors have nothing to report.

## Ethics Statement

The animal experiment was approved by the Ethics Committee of Shanghai General Hospital (Approval No. 2025AW229).

## Consent

The authors have nothing to report.

## Conflicts of Interest

The authors declare no conflicts of interest.

## Supporting information


**Figure S1:** Hypoxia/reperfusion‐induced ferroptosis in neurons can be reversed by Lip‐1.
**Figure S2:** Lip‐1 treatment improves SCIRI.
**Figure S3:** USP5 is highly expressed in neurons.
**Figure S4:** USP5‐KO does not affect spinal cord development in mice.
**Figure S5:** Overexpression of USP5 exacerbates ferroptosis after SCIRI and impairs functional recovery.
**Figure S6:** Impact of USP5 knockdown on c‐MAF expression in neurons under OGD/R and Erastin treatment.
**Figure S7:** Inhibition of c‐MAF expression reverses USP5‐induced ferroptosis in neurons.

**Table S1:** Antibodies.

## Data Availability

The data that support the findings of this study are available from the corresponding author upon reasonable request.
