# Supplementary material for: USP5‐C‐MAF Axis Regulates Autophagy‐Dependent Neuronal Ferroptosis in Spinal Cord Injury Therapeutics
Source: CNS Neurosci Ther. 2026 Jun 11;32(6):e70854. doi: 10.1002/cns.70854 (PMC13255003; doi:10.1002/cns.70854)
Supplement: Supplementary file 1 — Figure S1: Hypoxia/reperfusion‐induced ferroptosis in neurons can be reversed by Lip‐1. Figure S2: Lip‐1 treatment improves SCIRI. Figure S3: USP5 is highly expressed in neurons. Figure S4: USP5‐KO does not affect spinal cord development in mice. Figure S5: Overexpression of USP5 exacerbates ferroptosis after SCIRI and impairs functional recovery. Figure S6: Impact of USP5 knockdown on c‐MAF expression in neurons under OGD/R and Erastin treatment. Figure S7: Inhibition of c‐MAF expression reverses USP5‐induced ferroptosis in neurons. [file CNS-32-e70854-s001.docx]

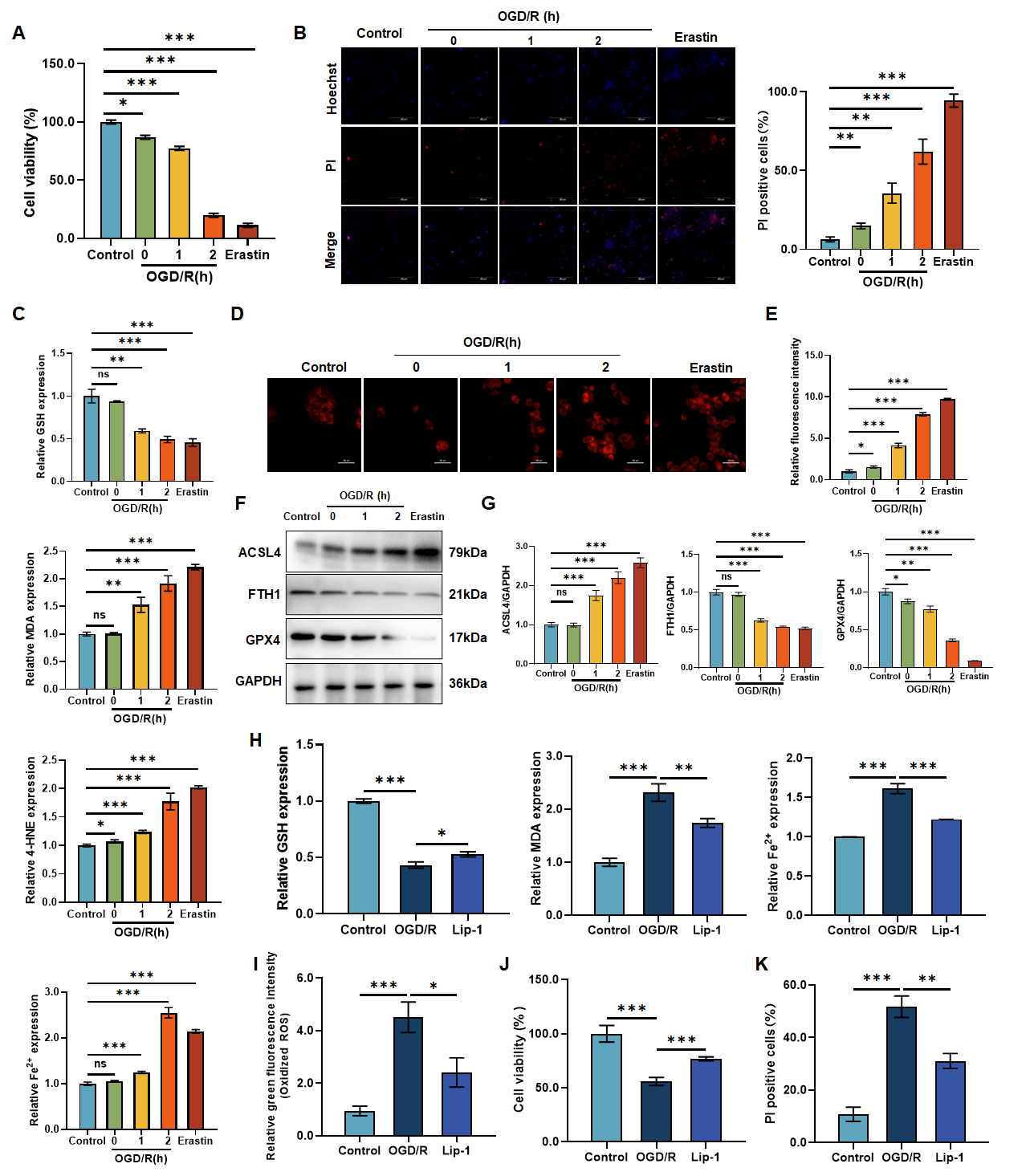
**Figure S1:** **Hypoxia/reperfusion-induced ferroptosis in neurons can be reversed by Lip-1**

(A) Cell viability of primary neurons after different durations of OGD/R treatment (0, 1, 2 h) or Erastin exposure, evaluated by CCK-8 assay.

(B) PI and Hoechst 33342 staining of neurons to assess cell membrane integrity following OGD/R or Erastin treatment. Quantification of PI-positive cells is shown on the right. Scale bar: 50 μm.

(C) Measurement of ferroptosis-related biochemical indicators, including GSH content, MDA levels, 4-HNE, and intracellular Fe²⁺ concentration using respective detection kits.

(D) Detection of intracellular reactive oxygen species (ROS) in neurons subjected to OGD/R or Erastin using a fluorescent probe. Scale bar: 100 μm.

(E) Quantification of ROS fluorescence intensity from panel (D) using ImageJ.

(F) Western blot analysis of ferroptosis-related proteins ACSL4, FTH1, and GPX4 in neurons after OGD/R or Erastin exposure. GAPDH was used as loading control.

(G) Densitometric quantification of protein expression from (F), normalized to GAPDH.

(H) Measurement of GSH, MDA, and Fe²⁺ levels in neurons treated with OGD/R with or without Lip-1 (100 nM).

(I) Quantification of ROS fluorescence intensity in the control, OGD/R, and Lip-1-treated groups.

(J) CCK-8 assay assessing the protective effect of Lip-1 on neuronal viability after OGD/R.

(K) PI staining quantification of neuronal death in the control, OGD/R, and Lip-1 groups.

The data are presented as the mean ± SEM. ns, p˃0.05, *p < 0.05, **p < 0.01, ***p < 0.001.


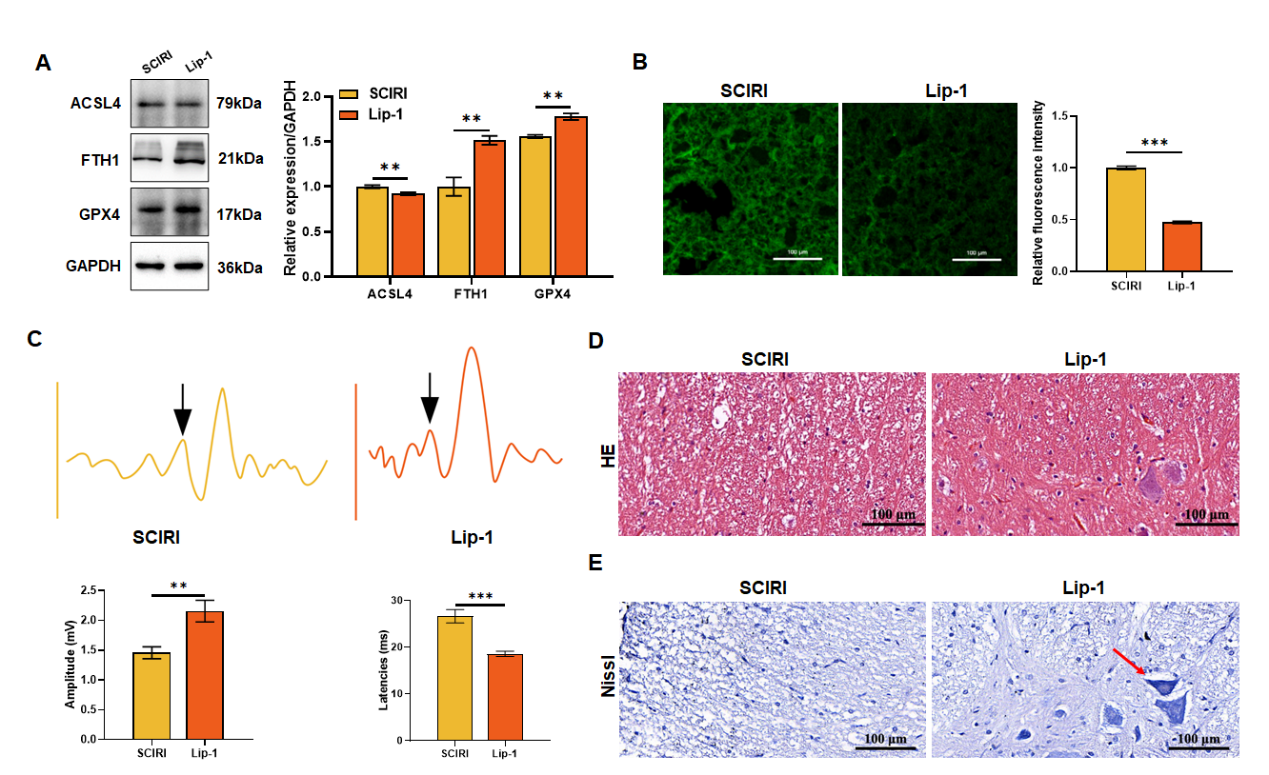


**Figure S2: Lip-1 treatment improves SCIRI**

(A) Western blot analysis of ferroptosis-associated proteins ACSL4, FTH1, and GPX4 in spinal cord tissues from SCIRI mice with or without Lip-1 treatment. GAPDH was used as loading control. Quantification is shown on the right.

(B) Representative fluorescence images showing reactive oxygen species (ROS) levels in spinal cord sections from SCIRI mice with or without Lip-1 administration. Scale bar: 100 μm. Quantification of fluorescence intensity is shown on the right.

(C) MEP recordings and corresponding quantification of amplitude and latency in SCIRI mice treated with or without Lip-1.

(D) HE staining of spinal cord tissues from SCIRI and Lip-1–treated mice. Scale bar: 100 μm.

(E) Nissl staining of spinal cord tissues from SCIRI and Lip-1–treated mice. Neurons are indicated by red arrows. Scale bar: 100 μm.

The data are presented as the mean ± SEM (n=3). *p < 0.05, **p < 0.01, ***p < 0.001.


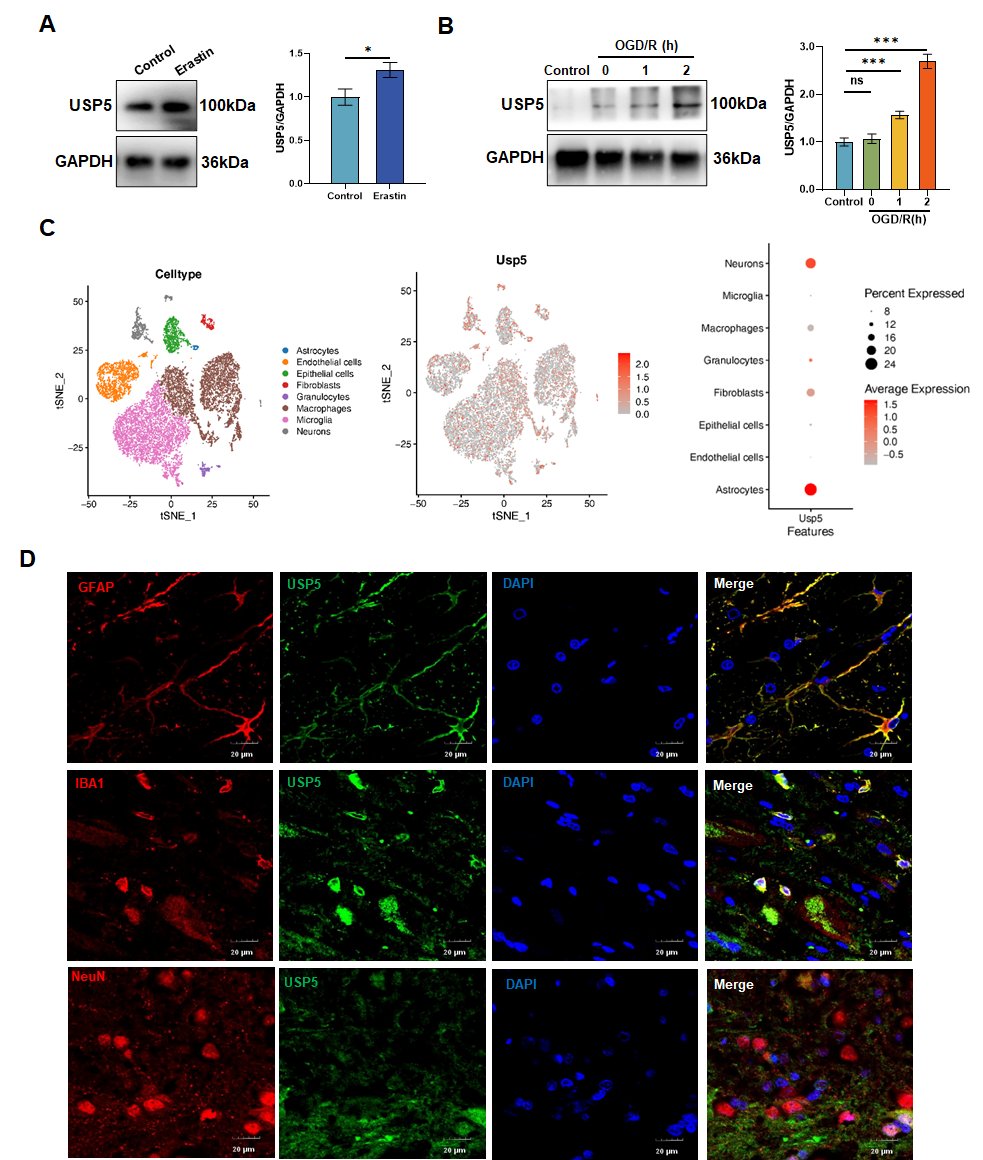


**Figure S3: USP5 is highly expressed in neurons**

(A) Western blot analysis of USP5 expression in neurons treated with or without Erastin. Quantification shown on the right. GAPDH served as loading control.

(B) Western blot analysis of USP5 expression in neurons subjected to OGD/R for different time points. Quantification shown on the right.

(C) Single-cell RNA-seq t-SNE clustering of spinal cord cells showing major cell types and USP5 expression patterns across cell populations.

(D) IF staining of spinal cord sections co-labeled with USP5 and lineage-specific markers: GFAP (astrocytes), IBA1 (microglia), and NeuN (neurons). Nuclei were stained with DAPI. Scale bars: 20 μm.

The data are presented as the mean ± SEM. ns, p˃0.05, *p < 0.05, ***p < 0.001.

**
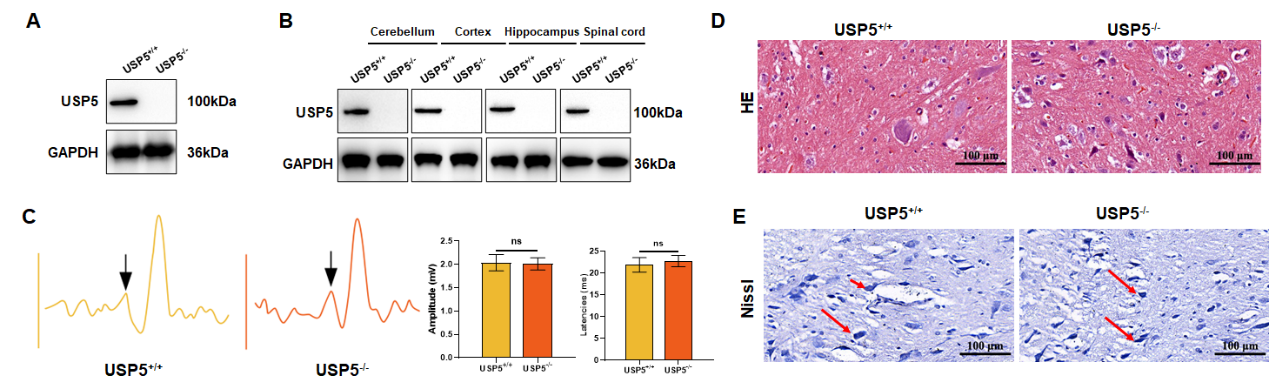
Figure S4: USP5-KO does not affect spinal cord development in mice**

(A) Western blot analysis of USP5 protein expression in primary neurons isolated from wild-type (USP5^⁺/⁺^) and knockout (USP5^⁻/⁻^) mice. GAPDH was used as the internal control.

(B) Western blot analysis of USP5 expression in different brain regions and spinal cord tissues harvested from USP5^+/+^ and USP5^-/-^ mice, including cerebellum, cortex, hippocampus, and spinal cord. GAPDH served as a loading control.

(C) Electrophysiological evaluation of motor nerve conduction in USP5^⁺/⁺^ and USP5^⁻/⁻^ mice. Compound muscle action potentials (CMAPs) were recorded to assess amplitude (left) and latency (right). Representative waveforms and quantitative analysis are shown.

(D) Representative HE staining of spinal cord sections from USP5^⁺/⁺^ and USP5^⁻/⁻^ mice. Scale bars: 100 μm.

(E) Representative Nissl staining images of spinal cord sections from USP5^⁺/⁺^ and USP5^⁻/⁻^ mice. Red arrows indicate Nissl bodies. Scale bars: 100 μm.

The data are presented as the mean ± SEM. ns, p˃0.05.

**
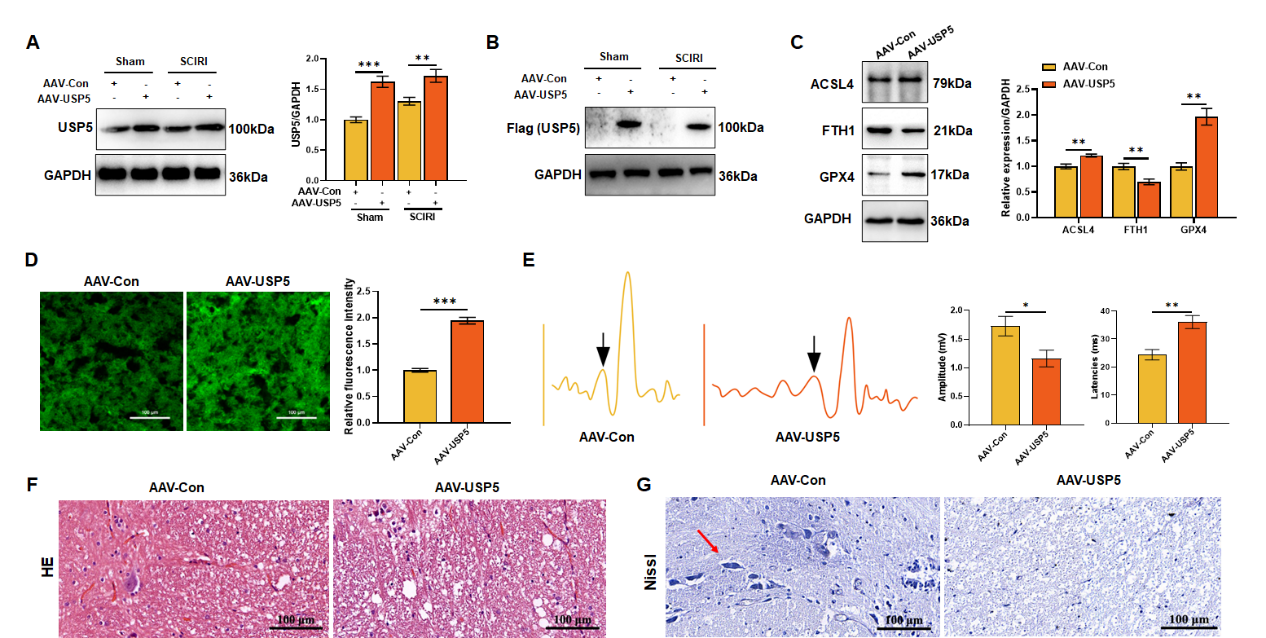
Figure S5: Overexpression of USP5 exacerbates ferroptosis after SCIRI and impairs functional recovery**

(A) Western blot analysis of endogenous USP5 protein levels in spinal cord tissues from mice injected with AAV-Con or AAV-USP5, under sham or SCIRI conditions. GAPDH was used as the internal control.

(B) Western blot detection of Flag-tagged USP5 protein in spinal cord tissues of mice treated or not treated with SCIRI, following AAV-mediated overexpression.

(C) Western blot analysis and quantification of ferroptosis-related proteins ACSL4, FTH1, and GPX4 in spinal cord tissues from AAV-Con and AAV-USP5 mice. GAPDH served as a loading control.

(D) Assessment of reactive oxygen species (ROS) levels in spinal cord tissues using fluorescence staining. Representative images and quantification of fluorescence intensity are shown. Scale bars: 100 μm.

(E) Electrophysiological evaluation of spinal nerve function via CMAPs recorded in AAV-Con and AAV-USP5 mice. Quantitative analysis of signal amplitude and latency is presented alongside representative traces.

(F) HE staining of spinal cord sections from AAV-Con and AAV-USP5 mice. Scale bars: 100 μm.

(G) Nissl staining of spinal cord tissues from AAV-Con and AAV-USP5 mice. Red arrows indicate Nissl bodies. Scale bars: 100 μm.

The data are presented as the mean ± SEM. ns, p˃0.05, *p < 0.05, **p < 0.01, ***p < 0.001.


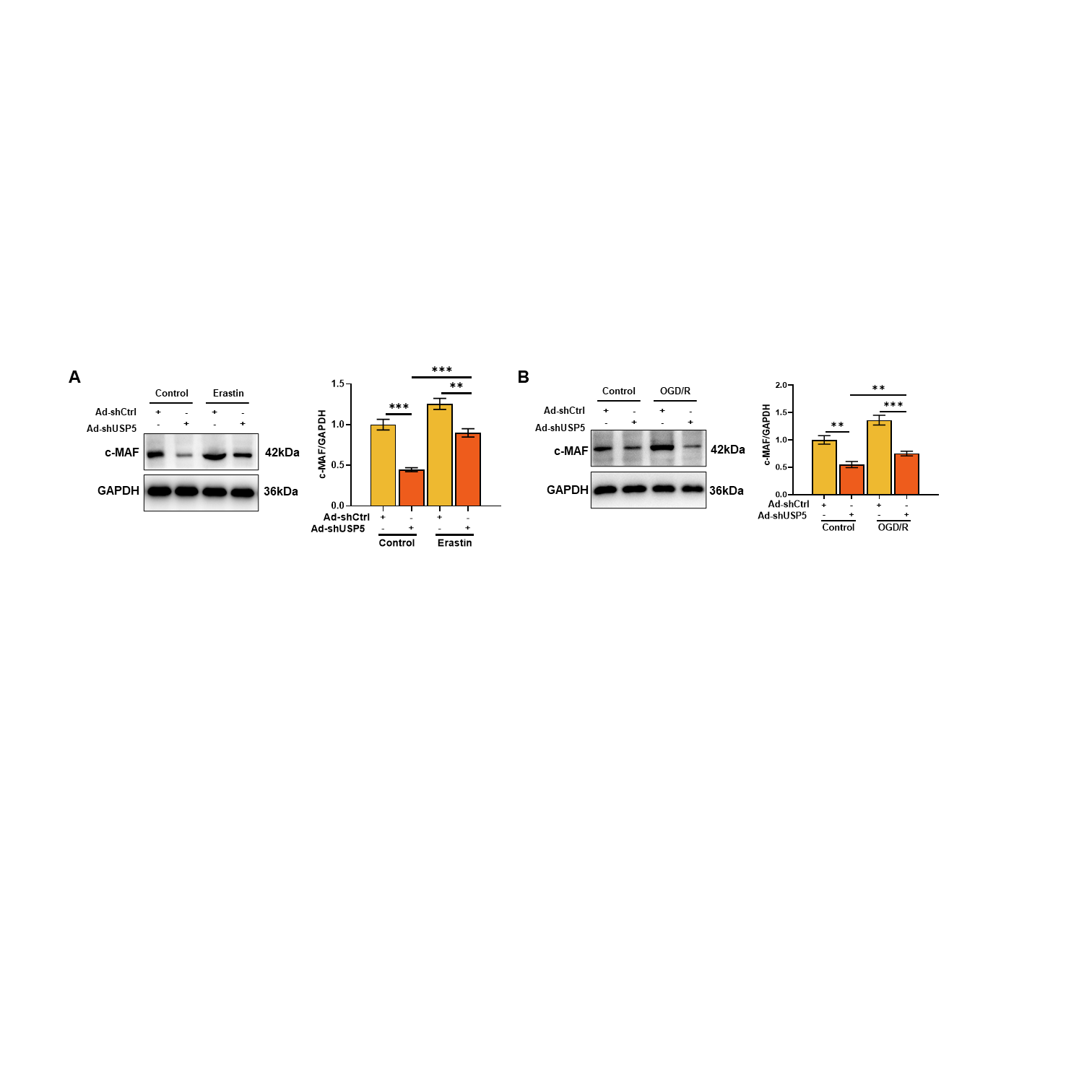


**Figure S6: Impact of USP5 knockdown on c-MAF expression in neurons under OGD/R and Erastin treatment**

(A) Western blot analysis of c-MAF protein expression in neurons infected with Ad-shCtrl or Ad-shUSP5, with or without Erastin treatment. GAPDH was used as the internal control.

(B) Western blot analysis of c-MAF protein levels in neurons subjected to OGD/R or control conditions, following infection with Ad-shCtrl or Ad-shUSP5.

The data are presented as the mean ± SEM. ns, **p < 0.01, ***p < 0.001.


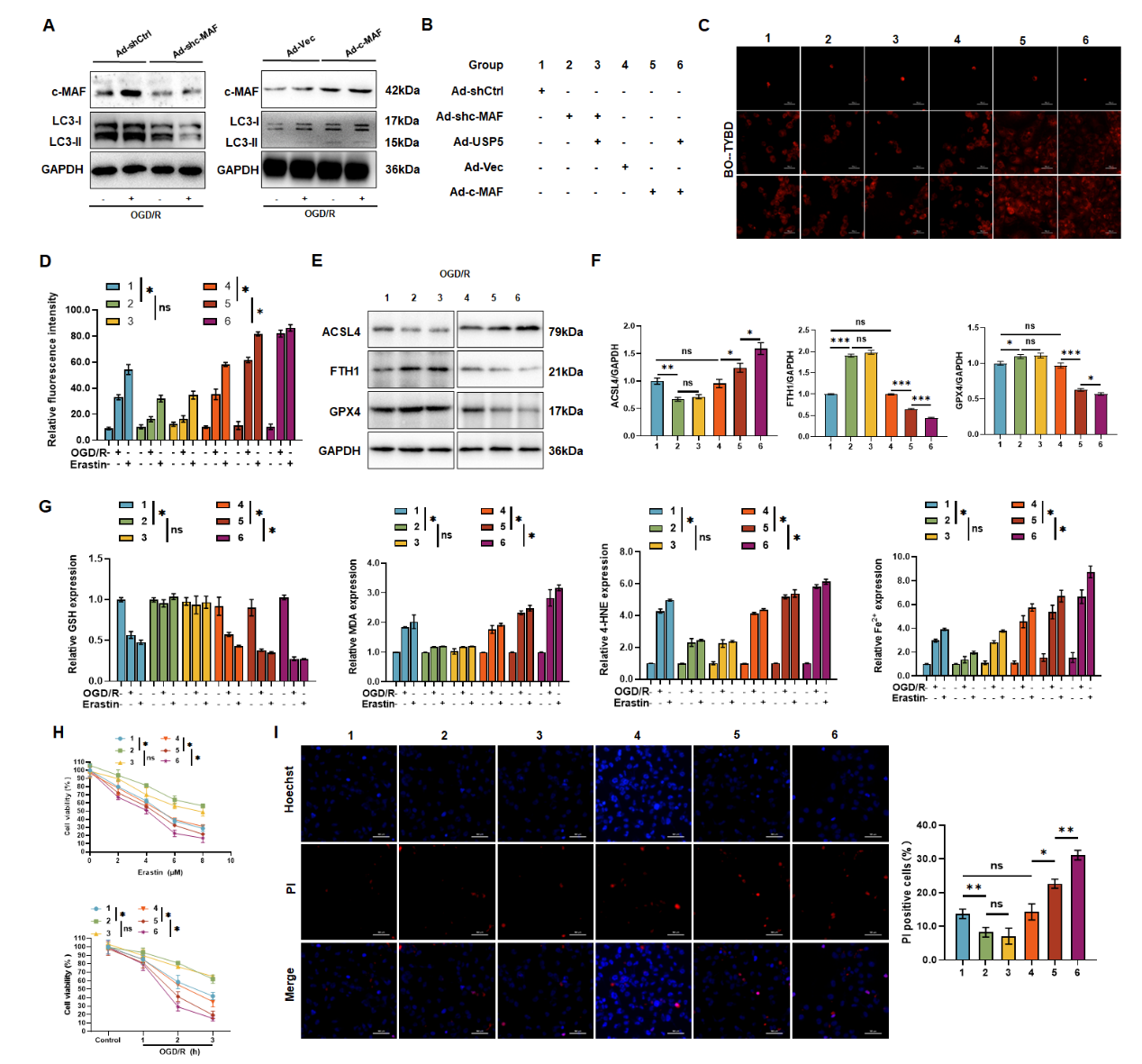


**Figure S7: Inhibition of c-MAF expression reverses USP5-induced ferroptosis in neurons**

(A) Western blot analysis of LC3-I and LC3-II protein expression in neurons with c-MAF knockdown or overexpression, under OGD/R treatment. GAPDH was used as a loading control.

(B) Experimental group assignments for the in vitro neuronal assays.

(C) Representative images of ROS levels detected by BODIPY™ 581/591 C11 staining in neurons from each group.

(D) Quantification of fluorescence intensity from panel C.

(E) Western blot analysis of ACSL4, FTH1, and GPX4 protein expression in neurons under different experimental conditions.

(F) Quantification of ACSL4, FTH1, and GPX4 protein expression levels shown in panel E.

(G) Relative levels of intracellular GSH, MDA, 4-HNE, and Fe²⁺ in neurons across the six experimental groups.

(H) Neuronal viability was assessed by CCK-8 assay under Erastin or OGD/R stimulation.

(I) Representative images of PI staining and quantification of PI-positive cells indicating neuronal death across experimental groups. Scale bars: 100 μm.

The data are presented as the mean ± SEM (n=3). *p < 0.05, **p < 0.01, ***p < 0.001.
